# Cardiac vagal activity is not a determinant of apnoea tolerance in trained breath‐hold divers

**DOI:** 10.1113/EP092998

**Published:** 2025-07-16

**Authors:** Benjamin S. Stacey, Giorgio Manferdelli

**Affiliations:** ^1^ Neurovascular Research Laboratory, Faculty of Life Sciences and Education University of South Wales Pontypridd UK; ^2^ Institute for Exercise and Environmental Medicine Texas Health Presbyterian Hospital Dallas Dallas Texas USA; ^3^ Department of Internal Medicine The University of Texas Southwestern Medical Center Dallas Texas USA

**Keywords:** apnoea, hypoxaemia, vagus

1

Once thought to be impossible, the physiological feat of enduring the compounding influence of hypercapnoea, hypoxaemia and hypertension during a voluntary breath hold for longer than a few minutes now stands at a breathtaking 11 min and 35 s. This capacity to sustain voluntary apnoea well beyond normative physiological limits is a hallmark of trained breath‐hold divers (BHD) within the extreme sport of free‐diving, in addition to the indigenous Japanese Ama and Philippine Bajau people of Asia who spend 50–60% of their working day underwater (Schagatay et al., 2011). This elite adaptation, often exceeding several minutes in duration, poses important questions regarding the autonomic, vascular and central control mechanisms that support such resilience to asphyxia. Of these mechanisms, little attention has been given to the contribution of the parasympathetic system, particularly cardiac vagal modulation of this unique apnoea tolerance.

As the largest cranial nerve, the vagus nerve exits the base of the skull bilaterally and innervates visceral organs in the neck, chest and abdomen, including the heart. Its afferent nerve fibres send visceral information to the central nervous system to modulate autonomic reflexes, including the arterial baroreflex, while its efferent fibres constitute the main cranial branch of the parasympathetic nervous system. While the activity of the vagus nerve has been widely investigated in animal models, in which the vagus nerve was surgically extracted and split into small strands until single‐fibre activity was detected (Paintal, 1953), and despite routine surgical procedures to stimulate the vagus nerve in human clinical populations, it was only recently that microneurography was used to investigate vagus nerve activity in human (Ottaviani et al., 2020). This is a welcome and much‐needed approach given that traditionally, we have relied on indirect and non‐specific metrics of sympathetic and parasympathetic modulation of the heart (i.e., heart rate variability). Briefly, following initial identification of the nerve via ultrasound (e.g., site and depth), a tungsten micro‐electrode is inserted through the skin at the posterior border of the sternocleidomastoid muscle and directed to the vagus nerve under ultrasound guidance (Figure 1). Vagus nerve microneurography can offer direct insights into the chronotropic and inotropic control of the heart, and given that parasympathetic nerves directed to the heart present a strong respiratory and cardiac modulation, vagus nerve microneurography may provide further insight into parasympathetic modulation of the arterial baroreflex and chemoreflex – an important consideration for those involved in physiological (exercise, sex differences), environmental (hypoxia and/or hyper‐ or hypocapnia, cold, heat) and clinical (hypertension, autonomic disorders) research.

**FIGURE 1:**
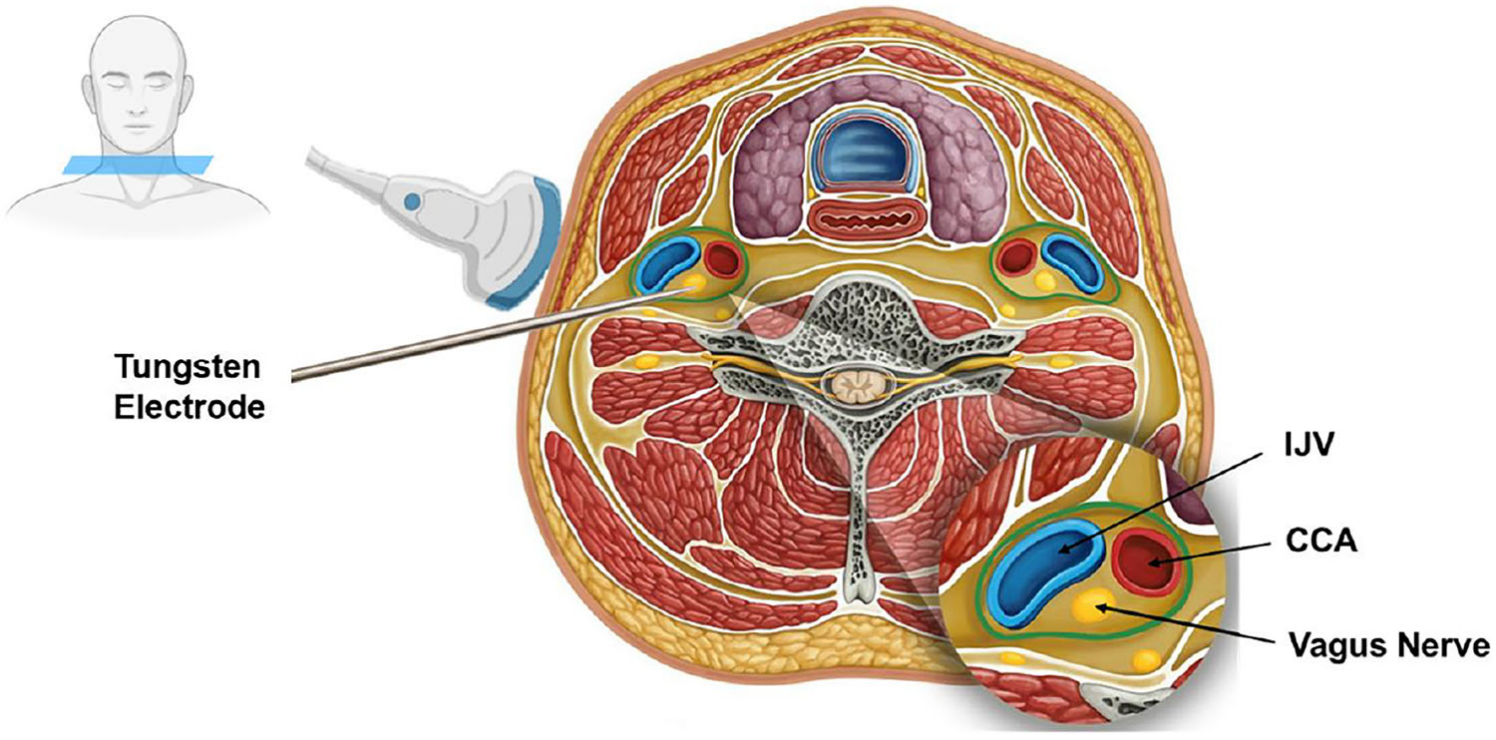
Schematic representation of vagus nerve microneurography. With the participant's head turned laterally, a tungsten electrode is inserted throughout the skin under ultrasound guidance into the vagus nerve located lateral to the common carotid artery (CCA) and posterior to the internal jugular vein (IJV). Axial representation at the level of the neck. Adapted from Ottaviani et al. (2020).

In this issue of *Experimental Physiology*, Macefield et al. (2026) employed vagus nerve microneurography to explore to what extent cardiac vagal modulation contributes to the remarkable apnoea tolerance observed in trained breath‐hold divers (BHD). Microelectrode recordings were taken at the right cervical vagus nerve in trained BHD (10 male; mean age: 36 ± 7 years) and 10 untrained controls (8 male, 2 female; mean age: 23 ± 3 years) to capture cardiac‐related vagal discharges. Participants were examined across a spectrum of respiratory challenges, including tidal breathing (5 min), slow‐deep breathing (5 breaths per min for 2 min), inspiratory‐capacity apnoea (maximally inflated lungs with a closed glottis for 40 s) and end‐expiratory apnoea (breath hold at functional residual capacity for 40 s). In the trained BHD, a maximal apnoea was attempted following a period of hyperventilation that excluded glossopharyngeal insufflation (i.e., lung packing, which increases pulmonary oxygen capacity and decreases arterial carbon dioxide) to avoid accidental dislodgement of the microelectrode.

Contrary to the study's initial hypothesis, no significant differences were observed for cardiac vagal activity between groups during any of the respiratory conditions. This was especially surprising for the maximal apnoea condition in which the average/longest maximal apnoea time in the trained BHD was 301/409 s andthat the involuntary breathing movements against a closed glottis occurred at around 128 s would have caused a large change in intrathoracic pressure affecting the firing of cardiac mechanoreceptors. These findings therefore suggest that cardiac vagal feedback may not be a key mediator underpinning this incredible apnoea tolerance and raise the question once again – What are the underlying mechanisms responsible for this astounding physiological feat?

As with many unknowns in human physiology, it can be insightful to turn our attention to living examples within the animal kingdom – in the context of anoxia tolerance, the crucian carp (*Carassius carassius*) leaves all others gasping for air. One particular reason for this tolerance is thought to be an adaptive, but reversible brain swelling (Wilkie et al., 2015), which likely also occurs during static apnoea in trained BHD, with evidence of a 59% greater cerebral blood flow and blood–brain barrier disruption at apnoea breakpoint, but in the absence of neuronal–parenchymal damage (Bain et al., 2018). Indeed, an increase in extracellular space could act to buffer a rise in interstitial concentrations of neurotransmitters (e.g., glycine and glutamate) that has the capacity to attenuate extracellular potassium and thus depolarise the brain (Bailey et al., 2009). Furthermore, under prolonged periods of anoxia, the crucian carp depresses its cardiac activity via decreased intrinsic heart rate and altered cardiac action potential characteristics, attenuating the energetic costs associated with ion pumping. Therefore, although cardiac activity via vagus nerve modulation may not be a primary determinant of this outstanding tolerance in BHD, other mechanisms involved in the intrinsic and/or autonomic control of heart rate might be involved.

In summary, the study by Macefield et al. (2026) provides compelling evidence that cardiac vagal modulation does not underlie enhanced apnoea tolerance of trained BHDs and encourages a redirection of research effort towards alternative mechanisms that may yield better insights into understanding this extreme human tolerance to hypoxaemia and hypercapnoea.

## AUTHOR CONTRIBUTIONS

Both authors have read and approved the final version of this manuscript and agree to be accountable for all aspects of the work in ensuring that questions related to the accuracy or integrity of any part of the work are appropriately investigated and resolved. All persons designated as authors qualify for authorship, and all those who qualify for authorship are listed. GM is supported by an American Heart Association postdoctoral fellowship (25POST1368578).

## CONFLICT OF INTEREST

None declared.
